# New Findings in a Global Approach to Dissect the Whole Phenotype of *PLA2G6* Gene Mutations

**DOI:** 10.1371/journal.pone.0076831

**Published:** 2013-10-09

**Authors:** Mustafa A. Salih, Emeline Mundwiller, Arif O. Khan, Abdulmajeed AlDrees, Salah A. Elmalik, Hamdy H. Hassan, Mohammed Al-Owain, Hisham M. S. Alkhalidi, Istvan Katona, Mohammad M. Kabiraj, Roman Chrast, Amal Y. Kentab, Hamad Alzaidan, Richard J. Rodenburg, Thomas M. Bosley, Joachim Weis, Michel Koenig, Giovanni Stevanin, Hamid Azzedine

**Affiliations:** 1 Division of Pediatric Neurology, College of Medicine, King Saud University, Riyadh, Saudi Arabia; 2 Institut du Cerveau et de la Moelle épinière (ICM), Genotyping and Sequencing Facility, Groupe Hospitalier Pitié-Salpêtrière (GHPS), Paris, France; 3 Division of Paediatrics Ophthalmology, King Khaled Eye Specialist Hospital, Riyadh, Saudi Arabia; 4 Department of Physiology, College of Medicine, King Saud University, Riyadh, Saudi Arabia; 5 Department of Radiology, College of Medicine, King Saud University, Riyadh, Saudi Arabia; 6 Department of Medical Genetics, King Faisal specialist Hospital & Research Centre, Riyadh, Saudi Arabia; 7 Department of Pathology, College of Medicine, King Saud University, Riyadh, Saudi Arabia; 8 Institut für Neuropathologie, Universitätsklinikum der RWTH, Aachen, Germany; 9 Department of Neurosciences, Armed Forces Hospital, Riyadh, Saudi Arabia; 10 Department of Medical Genetics, Faculty of Biology and Medicine, University of Lausanne, Lausanne, Switzerland; 11 Radboud University Medical Center, Nijmegen Center for Mitochondrial Disorders, Department of Pediatrics, Department of Laboratory Medicine, 774 Laboratory for Genetic, Endocrine and Metabolic disorders (LGEM), Nijmegen, The Netherlands; 12 Department of Ophthalmology, College of Medicine, King Saud University, Riyadh, Saudi Arabia; 13 Neurology Division, Cooper University Hospital, Camden, New Jersey, USA; 14 Institut de Génétique et de Biologie Moléculaire et Cellulaire (IGBMC), Centre National de la Recherche Scientifique (CNRS)/*Institut National de la Santé et de la Recherche Médicale* (INSERM)/*Université de Strasbourg, et Collège de* France, Illkirch, France; 15 École Pratique des Hautes Etudes (EPHE), Paris, France; 16 INSERM-U975, Paris, France; 17 CNRS-UMR (*Unité mixte de Recherche*) 7225, Paris, France; 18 *Université Pierre et Marie Curie* – Paris 6, (UMR-S)_975, *Centre de Recherche de l’Institut du Cerveau et de la Moelle épinière* (cricm), *Groupe Hospitalier Pitié-Salpêtrière* (GHPS), Paris, France; 19 *Assistance Publique des Hôpitaux de Paris* (APHP), *département de Génétique et Cytogénétique*, GHPS, Paris, France; Instituto de Ciencia de Materiales de Madrid - Instituto de Biomedicina de Valencia, Spain

## Abstract

Mutations in *PLA2G6* gene have variable phenotypic outcome including infantile neuroaxonal dystrophy, atypical neuroaxonal dystrophy, idiopathic neurodegeneration with brain iron accumulation and Karak syndrome. The cause of this phenotypic variation is so far unknown which impairs both genetic diagnosis and appropriate family counseling. We report detailed clinical, electrophysiological, neuroimaging, histologic, biochemical and genetic characterization of 11 patients, from 6 consanguineous families, who were followed for a period of up to 17 years. Cerebellar atrophy was constant and the earliest feature of the disease preceding brain iron accumulation, leading to the provisional diagnosis of a recessive progressive ataxia in these patients. Ultrastructural characterization of patients’ muscle biopsies revealed focal accumulation of granular and membranous material possibly resulting from defective membrane homeostasis caused by disrupted PLA2G6 function. Enzyme studies in one of these muscle biopsies provided evidence for a relatively low mitochondrial content, which is compatible with the structural mitochondrial alterations seen by electron microscopy. Genetic characterization of 11 patients led to the identification of six underlying *PLA2G6* gene mutations, five of which are novel. Importantly, by combining clinical and genetic data we have observed that while the phenotype of neurodegeneration associated with *PLA2G6* mutations is variable in this cohort of patients belonging to the same ethnic background, it is partially influenced by the genotype, considering the age at onset and the functional disability criteria. Molecular testing for *PLA2G6* mutations is, therefore, indicated in childhood-onset ataxia syndromes, if neuroimaging shows cerebellar atrophy with or without evidence of iron accumulation.

## Introduction

Neurodegeneration associated with *PLA2G6* mutations (PLAN) constitutes a heterogeneous group of clinical entities which encompasses infantile neuroaxonal dystrophy (INAD1/NBIA2A, MIM # 256600), atypical neuroaxonal dystrophy (NAD), idiopathic neurodegeneration with brain iron accumulation including Karak syndrome (NBIA2B, # MIM 610217) [1-9] and the recently reported syndrome of adult-onset dystonia-Parkinsonism (PARK 14, MIM # 612953) [9]. PARK 14 is characterized by rapidly progressive young-adult onset Parkinsonism associated with dystonia, cognitive decline and cerebral atrophy on MRI [9]. Onset may vary between 4 and 30 years, and abnormal brain iron may not be evident on MRI studies until late in the disease course [10-12].

INAD1/NBIA2A caused by mutations in *PLA2G6* gene starts typically between ages 6 months and 3 years with rapid progression of truncal hypotonia, progressive psychomotor delay, cerebellar ataxia, symmetric pyramidal tract signs and tetraparesis (usually spastic but sometimes areflexic). Children commonly manifest strabismus, nystagmus and optic atrophy, and lose the ability to walk shortly after attaining it or never learn to walk [4,6]. INAD is pathologically characterized by the presence of spheroids in both the central and peripheral nervous system [4,6,13].

Onset of atypical NAD is later than the one observed in INAD (about 4 years of age). Phenotype includes gait instability and delayed speech in >50% of patients, progressive dystonia and dysarthria, optic atrophy, tetraparesis (spastic or areflexic), and neurobehavioral disturbances. Neuroimaging reveals presence of cerebellar atrophy as early as at 2-3 years of age with no associated signs of iron deposition in the *globus pallidus*. Nevertheless, brain iron accumulation became obvious later and constitutes one of the diagnostic criteria [4]

Karak syndrome was described in two adolescent siblings with mutations in *PLA2G6* gene who suffered, since age 6 years, from slowly progressive ataxia associated with cognitive decline. Choreiform movements were evident by 9 years and ambulation was lost by 10 years [1]. Although both patients had normal motor power, pyramidal signs in addition to cerebellar and extrapyramidal signs were evident. On MRI, they had moderate degree of cerebellar atrophy, accumulation of iron in the *substantita nigra*, as well as a central hyperintensity within a region of hypointensity in the medial globus pallidus (the eye-of-the-tiger-sign).

*PLA2G6* gene encodes iPLA_2_-VIA, one of several calcium-independent phosphatases, which catalyzes the hydrolysis of glycerophospholipids, generating a free fatty acid, usually arachidonic acid, and a phospholipid [14]. Through altering the phospholipid composition of cellular and subcellular membranes, defects in iPLA2-VIA lead to failure to repair oxidative damage to membrane phospholipids and adversely affect membrane fluidity, permeability or iron homeostasis. This results in structural damage of the membrane and neuronal apoptosis, which may underlie the axonal pathology and brain iron accumulation [14,15].

Here we report the clinical, electrophysiological, neuroimaging, neuropathological and genetic features of 11 patients from 6 consanguineous families of ethnic Arab background suffering from NBIA syndromes associated with *PLA2G6* gene mutations, and followed for a maximum period of 17 years.

## Patients and Methods

### Ethics Statements

Clinical evaluation, blood and muscle biopsy samples were obtained specifically for this study after a written informed consent, as defined by the local Ethics Committee-Deanship of Scientific Research College of Medicine Research Center (CMRC), King Saud University, Riyadh, according to the principles of the declaration of Helsinki. The ethics committee specifically approved this study.

### Clinical investigations

Eleven patients (5 males and 6 females) from 6 consanguineous Saudi Arabian families were studied. Ten of these were evaluated at the Division of Pediatric Neurology, King Khalid University Hospital, and College of Medicine, King Saud University, Riyadh, from October 1993 to February 2011. One patient was seen and investigated at King Faisal Specialist Hospital and Research Centre, Riyadh. All patients were examined by a neurologist, an ophthalmologist and a neuro-ophthalmologist to document clinical status at the time of enrolment.

### Neurophysiological studies

Standard EEG recordings were done for three patients who had epilepsy, and another two who were asymptomatic. Nerve conduction studies were performed and recorded in all patients except one (F4 [P1]), following a conventional protocol [16] using surface electrodes and stimulator. Age-appropriate reference values were utilized for interpretation [17-19]. Motor conduction study in the upper limbs was done for the median nerve in 7 and for the ulnar nerve in 2 patients, while it could not be performed in one patient (F1 [P1]), because of the presence of bedsores and contracture deformities.

### Neuro-ophthalmic and neuroimaging examinations

Ophthalmologic examinations were occasionally limited because many of the patients were young and because of the cognitive complications of the neurodegenerative disorder. The assessment of optic disk pallor was done using indirect ophthalmoscopy. All patients had visual evoked responses (VEPs) using flashing lights and P100 was the most important wave to identify. All patients had brain computer tomography (CT) and/or magnetic resonance imaging (MRI) [Siemens 1.5 Tesla].

### Structural and functional muscle analysis

Muscle biopsy (from the *vastus lateralis*) was undertaken in 4 patients and examined by standard histological techniques. Two glutaraldehyde-fixed muscle specimens were available for electron microscopy (EM) examination and processed as described by Weis et al. [20]. The quantification of intermyofibrillar mitochondria was done manually on 10 digital electron micrographs taken of randomly selected fields of the specimens with the magnification of 6000X [21]. In one biopsy, mitochondrial enzyme activities were measured in crude muscle extracts as described [22,23].

### Other investigations

Prior to the molecular diagnosis, detailed investigations were undertaken to rule out inborn errors of metabolism, progressive ataxia syndromes and/or mitochondrial disorders, and all were within normal limits. These tests included measurements for renal, hepatic, thyroid functions, creatine-phosphokinase, blood glucose, ammonia, lactate, pyruvate, Tandem metabolic screen, biotinidase, triglycerides and cholesterol, isoelectric focusing of transferrin, very long chain fatty acids, serum copper and ceruloplasmin, serum vitamins E and B12, ∝-fetoprotein and carcinoembryonic antigen, antinuclear antibodies, karyotype, bone marrow aspirate, urinary amino acids, organic acids and sulfocysteine-xanthine, and CSF glucose, proteins and lactate. Because ataxia was the earliest presenting clinical sign, prior to start genotyping of the families, two patients have had molecular investigations for Friedreich ataxia and no abnormal GAA expansion was found in *FXN* gene (data not shown).

### Genetic and bioinformatics analysis

Genotyping of 5 out of 6 families was performed using 10 microsatellite markers ([D20S: 842, 181, 867, 116, 482 and 8959] and [D22S:1177, 1045, 272 and 428]) flanking the *PANK2* and *PLA2G6* genes, respectively. PCRs were performed using classical procedures. The PCR products were analysed on an ABI 3730 automatic sequencer (Applied Biosystems). The genotypes were determined using GeneMapper version 4 software (Applied Biosystems) and the haplotypes were reconstructed manually. Allegro software [24] was used for multipoint LOD scores calculations. Mutation analysis in the *PLA2G6* gene was done by specific PCR amplification of all exons and their flanking intron segments (primers and annealing conditions available upon request) in a Veriti thermal cycler (Applied Biosystems) followed by direct sequencing with Big Dye terminator chemistry in an ABI3730 sequencer, according to the manufacturer’s recommendations (Applied Biosystems). Sequences were analysed using Seqscape 2.6 software (Applied Biosystems). In addition, 86 to 175 North African unrelated healthy subjects were screened to evaluate the frequency of the mutations. Gene rearrangements in *PLA2G6* and *PANK2* were analyzed using the specific Multiplex Ligation-dependent Probe Amplification (MLPA) kit # P120 (MRC-Holland) with the ABI3730 sequencer and analysis of the electrophoretic profiles with GeneMapper 4 (Applied Biosystems). Deleterious defects of missense variants were analyzed with the SIFT, PolyPhen2 (http://genetics.bwh.harvard.edu/pph) Multiple alignments of orthologs of *PLA2G6* for the analysis of the conservation of affected amino acids in various species were done with PolyPhen2.

## Results

### Symptoms and signs

All affected individuals had normal perinatal history and early developmental milestones within the first 9 months of life. Three children (F1 [P1], F2 [P1 and P2]) [Table 1] never walked; but the other 8 were able to walk at a mean age of 13 months (median 12 months, range 12-16 months). Ataxia was the earliest appearing symptom (Table 1) at a median age of 14 months (mean=29.6 months, range 9 months-6 years). This was followed by motor and cognitive decline in all patients, but with varying pace. Except for the 3 patients who never walked and another (F6 [P2], Table 1) who was still walking until age 20 years and lost ambulation at 20.5 years, all other patients became wheelchair-bound at a median age of 6 years (mean=9.5 years, range 3-17 years). Psychiatric symptoms were evident in 5 (45%) patients. Two siblings (F6 [P2 and P1]) had episodes of phobias and panic attacks, a third child (F6 [P4]) experienced bouts of rage at home, the forth (F1 [P1]), had episodes of self-mutilation, whereas the fifth (F2 [P2]) had bouts when he became frightened. One patient [F6 (P1)] developed complex partial seizures at the age of 21 years, and was well-controlled with lamotrigine. Two siblings (F2 [P1 and P2]) had seizures starting at 18 months. These were infrequent, brief (less than 1 minute) and consisted of blue spells associated with body stiffness.

**Table 1:** Clinical features of 11 patients with *PLA2G6* gene mutations.

	**Family (Patient No.)**										
	**F1 (P1)**	**F2 (P2)**	**F2(P1)**	**F3 (P2)**	**F3 (P1)**	**F4 (P1)**	**F5 (P1)**	**F6 (P3)**	**F6 (P4)**	**F6 (P1)**	**F6 (P2)**
Gender	F	M	F	M	F	F	M	M	M	F	F
Age at last assessment	9y 9mo	2y 5 mo	4y 2mo	5y	10y	3y 6mo	10y	21y	19y	27y	20y
Age at death (Cause)	Alive	Alive	Alive	Alive	12y 10 mo (H1N1 swine flu)	Alive	Alive	Alive	Alive	28y (Septicemia following bed sores)	Alive
Age at walking	NWI	NWI	NWI	1 y	15 mo	14 mo	1y	16 mo	1y	1y	1y
Ataxia (onset)	9 mo	1y	1y	Since walking	Since walking	Since walking	Since walking	3y	5y	6y	6y
History of leg stiffness	None	None	None	Yes	None	Yes	Yes	None	None	Yes	Yes
Cognitive decline	Yes	Yes	Yes	Yes	Yes	Yes	Yes	Yes	Yes	Yes	Yes
Psychiatric symptoms	Yes (episodesof self mutilation)	Yes (episodes of being frightened)	None	None	None	None	None	None	Yes (bouts of rage)	Yes (phobias, panic attacks)	Yes (phobias, panic attacks)
Clinical seizures (onset)	None	Yes (18mo)	Yes (18mo)	None	None	None	None	None	None	Yes (21y)	None
Wheelchair-bound (onset)	NW**I**	NWI	NWI	Yes (3y)	Yes (3y)	Yes (3y 6mo)	Yes (6y)	Yes (17y)	Yes (17y)	Yes (17y)	yes (20.5y)
Microcephaly	Yes	None	None	None	Yes	Yes	None	None	None	None	None

**NWI** = Never walked independently

Physical examination [Table 2] revealed microcephaly (head circumference <2SD) in 3 (27%) patients and strabismus was evident in 5 (45%). Optic nerve pallor was seen in 9 (82%) and was associated with strabismus in 4 (36%). All patients showed nystagmus or saccadic pursuit. When assessed at a median age of 10 years (mean = 12.0 years; range 2.4-27 years), leg stiffness was apparent in 5 (45%) patients and 9 (82%) showed hyperreflexia of the upper and/or lower limbs. Extrapyramidal signs in the form of facial dyskinesia, dystonia, bradykinesia and/or rigidity were observed in 7 (64%) patients. Contracture deformities of the limbs, including heelcord tightening and *equinovarus*, were ascertained in 8 (73%), and 2 (18%) patients had severe scoliosis. Other complications which developed over the years included multiple bed sores and osteomyelitis in a 27-year-old female (F6 [P1]), and malnutrition (requiring feeding through gastrostomy tube) and bed sores in another girl aged 9.8 years (F1 [P1]). Two patients died at ages of 12.8 years (F3 [P1]) and 28 years (F6 [P1]). The causes of death were, respectively, respiratory infection due to H1N1 swine flu and septicaemia following bed sores.

**Table 2:** Clinical features of 11 patients with *PLA2G6* gene mutations.

	**Family (Patient No.)**										
	**F1 (P1)**	**F2 (P2)**	**F2 (P1)**	**F3 (P2)**	**F3 (P1)**	**F4 (P1)**	**F5 (P1)**	**F6 (P3)**	**F6(4)**	**F6 (P1)**	**F6 (2)**
Strabismus	Yes	None	None	None	None	Yes	Yes	None	None	Yes	Yes
Optic nerve pallor	Yes (OU)	None	Yes (OU)	Yes (OU)	Yes (temporal OU)	Yes (OU)	None	Yes (OU)	Yes (temporal OU)	Yes (OU)	Yes (OU)
Nystagmus / saccadic pursuit	Yes	Yes	Yes	Yes	Yes	Yes	Yes	Yes	Yes	Yes	Yes
Extrapyramidal signs	UL (NA) LL (None)	None	Rigidity throughout elbow movement	Mild rigidity (UL)	Dystonia, facial dyskinesia, rigidity	None	Bradykinesia (UL)	Slight rigidity	None	Dystonia, facial dyskinesia, rigidity	Rigidity
Deep tendon reflexes	Brisk (UL)	Brisk (UL + LL)	Brisk (UL and knees) Present (ankles)	Brisk (UL + knees)	Brisk (UL)	Brisk (UL + LL)	Brisk (UL + knees)	Absent (UL)	Absent (UL)	Brisk (UL + knees)	Brisk
	Absent (LL)			Absent (ankles)	Absent (LL)			Absent (LL)	Absent (LL)	Absent (ankles)	(UL + knees) Absent (ankles)
Sensation (crude touch)	N	N	N	N	N	N	N	N	N	N	N
Contracture deformities	Multiple flexion contracture deformities (UL + LL) Severe scoliosis	None	Equinovarus	Heel cord tightening	Equinovarus Severe scoliosis	None	Heel cord tightening	None	Equinovarus	Equinovarus	Equinovarus
Other complications (surgical operations)	Malnutrition bed sores (Gastrostomy tube fixed)		(Tonsillectomy & adenoidectomy at 2y because of snoring at night)	None	None	None	None (Medial hamstring and tendoachilis release bilaterally)	None	None	Multiple bed sores osteo-myelitis	None

### Visual evoked potential (VEP) and electroencephalography (EEG)

Absent or delayed evoked potentials (VEP) were seen in 5/11 (45%) patients (Table 3). In the patient who developed clinical seizures at the age of 21 years (F6 [P1]), EEG showed normal background activity associated with frequent sharp, spike and slow-wave complexes seen on the left anterior frontal region. The elder of the two siblings with clinical seizures (F2 [P1]) had normal EEG; whereas the younger one (F2 [P2]) had non-specific generalized slow activity, more on the occipital electrodes. Another asymptomatic child (F4 [P1]), had excessive fast beta activity associated with predominantly right occipital and generalized epileptiform discharges. Diffuse fast activity over both hemispheres was seen in a third child (F1 [P1]) who had no clinical seizures.

**Table 3:** Neurophysiological tests and muscle biopsy characterization.

		**Family (Patient No.)**																				
		**F1 (P1)**		**F2 (P2)**	**F2 (P1)**		**F3 (P2)**		**F3 (P1)**	**F4 (P1)**		**F5 (P1)**		**F6 (P3)**		**F6 (P4)**		**F6 (P1)**			**F6 (P2)**	
Gender		F		M	F		M		F	F		M		M		M		F			F	
NCS (Table 4)		Axonal neuropathy		Axonal neuro-pathy	Axonal neuro-pathy		Axonal neuro-pathy		Axonal neuropathy	ND		Essentially Normal		Axonal neuropathy		Axonal neuropathy		Axonal neuropathy			Axonal neuropathy	
VEP (flash)		Normal		No response (OU)	No response (OU)		Delayed P100 (R & L)		Normal (R) Delayed P100(L)	Normal		Normal		Normal		Normal		No response (R) Delayed P100 (L)			Normal	
EEG		Diffuse fast activity over both hemi-spheres		*	WNL		ND		ND	**		ND		ND		ND		Frequent sharp, S&SLW complexes on L anterior frontal		ND		
**Muscle biopsy**	Histochemistry	Non-specific neurogenic changes	ND			Non-specific neurogenic changes		ND	ND		ND		ND		ND		ND		Non-specific neurogenic changes			Non-specific neurogenic changes
	Electron Microscopy	***	ND			ND		ND	ND		ND		ND		ND		ND		§			ND
	Bio-chemistry	ND	ND			ND		ND	ND		ND		ND		ND		ND		£			ND

Abbreviations: EEG = electroencephalogram; F = female; L = left; LL = lower limbs; M = male; mo=months; N = normal; NCS = nerve conduction studies; ND = not done; OU = each eye; P = patient; R = right; S = spike; SLW = slow wave; UL = upper limbs; VEP = visual evoked potentials; WNL = within normal limits; y = year* Non-specific generalized slow activity at 4-5y Hz with higher voltage, more on the occipital electrodes (during sleep). ** Excessive fast beta activity. Predominantly R occipital & generalized epileptiform discharges. ***Moderately increased lipid droplets. Subsarcolemma accumulation of membranous material. § Enlargement of the sarcoplasmic space between myofibrils associated with focal increase in granular and membranous material. £ Relatively low mitochondrial content.

### Nerve conduction studies

Distal axonal-type sensorimotor neuropathy was evident in 9 (90%) patients who had nerve conduction studies (NCS, [Tables 3 and 4]). Motor nerve conduction velocities (MNCVs) in the upper limbs were normal in 7 (78%) out of 9 tested patients and reduced in 2 (22%) [mean = 55.2± 9.12 m/s]. In the lower limbs, MNCVs were normal in 8 (80%) and unobtainable in 2 (20%) patients (mean = 49.25 ± 6.81 m/s, Table 4). In the upper limbs, the distal motor nerve latencies (DML) were within the normal range in all the 9 tested patients (100%) while those of the peroneal, done in 10 patients, were absent in 2 (20%) and prolonged in 3 (30%) patients.

**Table 4:** Nerve conduction studies in 10 patients with *PLA2G6* mutations.

	**Family (Patient No.)**		MNCV (m/s) [Normal values*]			Distal latency m/s [Normal values*]			CMAPs (mV) [Normal values*]			Sensory amplitude (µV) (SNAPs) [Normal values*]	
		**Age**	**Median**	**Ulnar**	**Peroneal**	**Median**	**Ulnar**	**Peroneal**	**Median**	**Ulnar**	**Peroneal**	**Median**	**Sural**
	**F1 (P1)**	9.8 y	-	-	41 [48.3±3.9]	-	-	9.7↑ [3.77±0.86]	-	-	0.5↓ [5.1±2.3]	-	12↓ [23.7±3.8]
	**F2 (P2)**	2.4 y	52 [52.71±3.71]	-	55 [51.21±3.95]	2.3 [1.89±0.17]	-	2.2 [2.57±0.4]	3.6↓ [5.96 ±2.01]	-	1.9↓ [4.25±1.59]	42 [12.02 ± 5.89]	11↓ [23.27±6.84]
	**F2 (P1)**	4.2 y	59 [56.48±2.36]	-	43 [53.21±3.95]	2.7 [2.03±0.25]	-	2.2 [3.02±0048]	3.8↓ [6.96 ±2.33]	-	1.6↓ [3.78±1.23]	68 [14.04 ± 5.99]	8↓ [22.66±5.42]
	**F3 (P2)**	5 y	52 [56.48±2.36]	-	60 [53.21±3.95]	2.7 [2.03±0.25]	-	2.9 [3.02±0048]	7.6 [6.96 ± 2.33]	-	2.2↓ [3.78±1.23]	38.4 [14.04 ± 5.99]	18.3 [22.66±5.42]
	**F3 (P1)**	10 y	57 [57.7±4.9]	-	54 [48.3±3.9]	3.1 [3.49±0.34]	-	3.9 [3.77±0.86]	2.7↓ [7.00±3.00]	-	2.6↓ [5.1±2.3]	82.1 [38.5±15.6]	25.8 [23.7±3.8]
	**F5 (P1)**	10 y	69 [57.7±4.9]	-	51 [48.3±3.9]	3 [3.49±0.34]	-	2.4 [3.77±0.86]	7.1 [7.00±3.00]	-	4.8 [5.1±2.3]	46.8 [38.5±15.6]	17.2↓ [23.7±3.8]
	**F6 (P3)**	21 y	-	67 [58.7±5.1]	47 [48.3±3.95]	-	3.2 [2.59±0.39]	6↑ [3.77±0.86]	-	2.9↓ [5.7±2.0]	1.2↓ [5.1±2.3]	50 [38.5±15.6]	19 [23.7±3.8]
	**F6 (P4)**	19 y	-	55 [58.7±5.1]	43 [48.3±3.9]	-	2.2 [2.59±0.39]	5.8↑ [3.77±0.86]	-	2.9↓ [5.7±2.0]	1.3↓ [5.1±2.3]	42 [38.5±15.6]	22 [23.7±3.8]
	**F6 (P1)**	27 y	43↓ [57.7±4.9]	-	NR [48.3±3.9]	2.6 [3.49±0.34]	-	NR [3.77±0.86]	3.3↓ [7.00±3.00]	-	NR [5.1±2.3]	24 [38.5±15.6]	NR [23.7±3.8]
	**F6 (P2)**	20 y	43↓ [57.7±4.9]	-	NR [48.3±3.9]	2.7 [3.49±0.34]	-	NR [3.77±0.86]	9.5 [7.00±3.00]	-	NR [5.1±2.3]	18.7↓ [38.5±15.6]	NR [23.7±3.8]

* Normal values of NCVs are age dependent and were taken from Ref.: 17, 18 and 19.

Abbreviations: = not done, CMAPs = compound muscle action potentials; EEG=electroencephalogram; L = left; MNCV = motor nerve conduction velocity, mV = millivolts, µV = microvolts, NR = not recordable; OU=each eye; R=right; S=spike; SLW=slow wave;;

y = year. - not done, ↑ increased value; ↓ decreased value.

The compound muscle action potentials (CMAPs) morphology was diphasic and the peak amplitudes were recorded. With the exception of one (F5 [P1]), all patients had either unobtainable or low amplitude CMAPs for the peroneal nerve, recorded from the extensor digitorum brevis (EDB) muscle; while in the upper limbs 3/9 patients (33%) showed normal CMAP amplitudes. The median sensory nerve action potentials (SNAPs) recorded in 9 patients were normal in 7 (77%) and reduced in one (F6 [P2]), while sural SNAPs were within the normal range in 4/10 (40%) patients.

### Neuroradiological features

**Figure 1:**
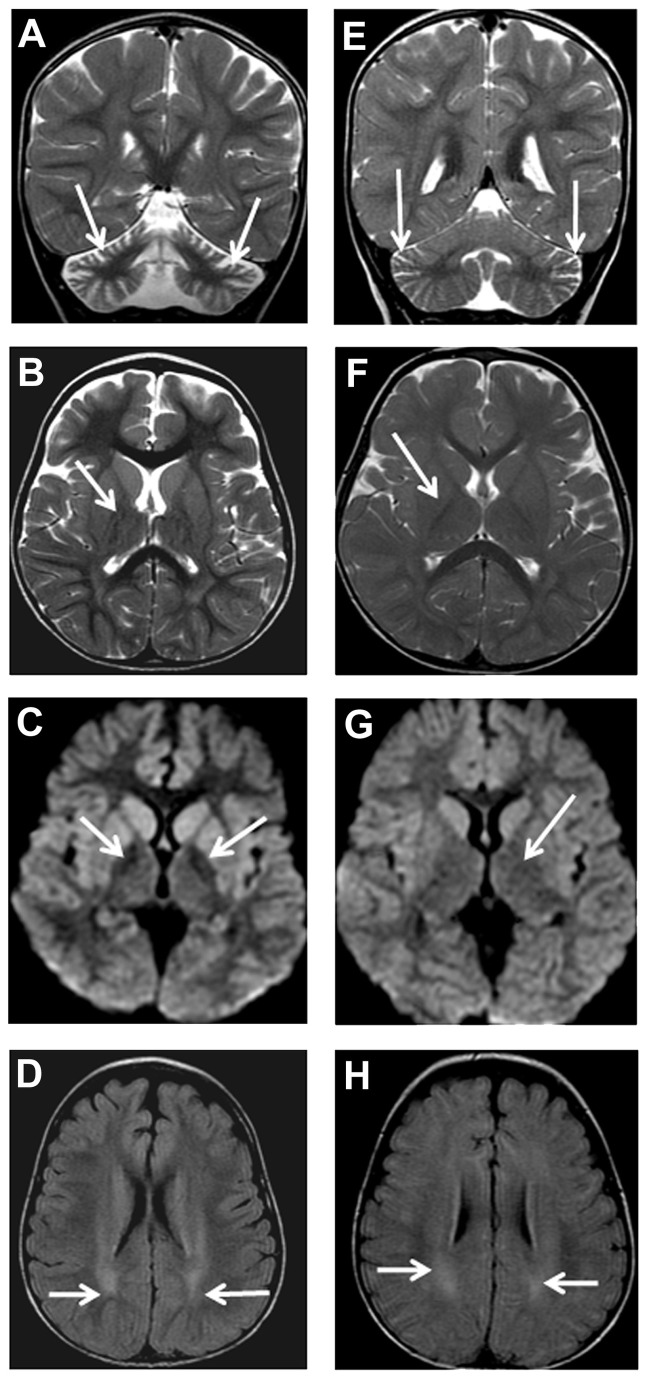
Patient F2 (P1) MRI, age 3 years 3 months (A-D). Coronal T2-weighted (A), axial T2-weighted (B), axial diffusion (C), axial FLAIR (D) showing cerebellar atrophy (arrows) with widening of cerebellar folia (A), T2 normal signal intensity (arrows) of *globus* *pallidus* (B), highlighted subtle iron deposition at globus pallidi (arrows) as reduction in signal intensity (C), and high signal intensity (arrows) at cerebral white matter (D). Patient F2 (P2) MRI, age 1 year 3 months (E-H)). Coronal T2-weighted (E), axialT2-weighted (F), axial diffusion (G), axial FLAIR (H) showing mild cerebellar atrophy (arrows) with mild widening of cerebellar folia (E), normal signal intensity (arrows) of *globus* *pallidus* (F and G), and high signal intensity (arrows) at cerebral white matter (H).

**Figure 2:**
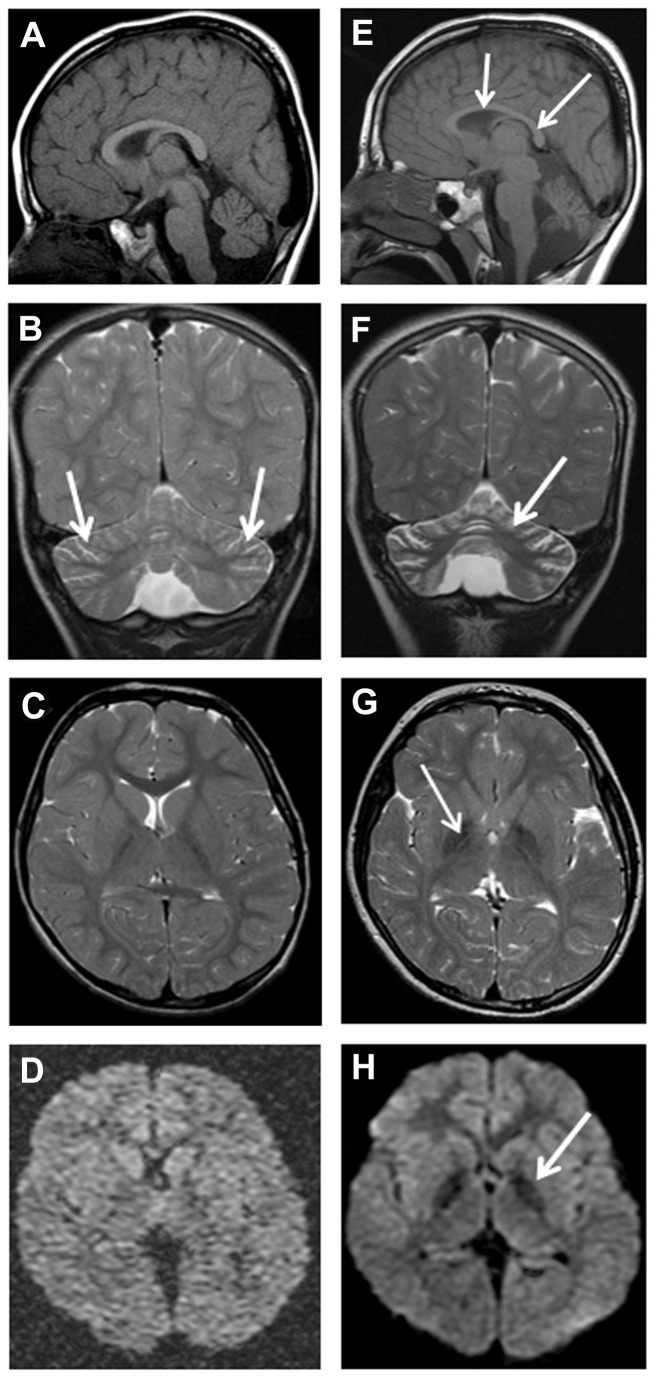
Patient F5 (P1) MRI, age 4 years 2 months (A-D), and 7 years 6 months (E-H). Sagittal T1-weighted (A and E), coronal T2-weighted (B and F), axial T2-weighted (C and G) and axial diffusion (D and H) sequences showing mild cerebellar cortical atrophy with mildly prominent folia (arrows in C). There is also simple corpus callosum (arrows in E). (F) Coronal T2-weighted reveals progressive cerebellar cortical atrophy and gliosis, with widening of folia and increased signal in the residual cerebellar cortex (arrow). Axial T2-weighted (G), and axial diffusion (H) sequences highlight iron deposition as reduction in signal intensity in the globus pallidi (arrows).

Brain imaging (Table 5) was undertaken in all patients and consisted of MRI in 9 (82%), CT in one (F6 [P4]) and CT and MRI in another patient (F1 [P1]). The first neuroimaging was performed at a median age of 4.3 years (mean = 7.9 years; range 1.3-19 years). Four (36%) patients had repeated MRI. Cerebellar cortical atrophy was universally present even at the age of 1.3 year (Figure 1). Of the 9 initial MRI scans with suitable quality, 6 (67%) revealed cerebellar cortical gliosis at a median age of 7.3 years (mean =7 years; range = 2.8-11.3 years). This manifested as high signal on either T2-weighted or fluid-attenuated inversion recovery (FLAIR) (Figure 2).

**Table 5:** Neuroimaging findings in 11 patients with *PLA2G6* mutations.

Family (Patient No.)	Scan type	Age	Cerebellar atrophy		Cerebellar gliosis	Low signal/density (globus pallidus)	Low signal/density(substantia nigra)	Cerebral white matter	Corpus callosum
F1 (P1)	MRI	3y 1 mo		+	+	+ (DWI)	+	-	-
	CT	4y 2 mo		+	1	-	-	-	I
	MRI	4y 2 mo		+	+	+ (T2, DWI)	+	-	S
F2 (P2)	MRI	1y 3 mo		+	-	-	-	H	-
F2 (P1)	MRI	3y 3 mo		+	-	+ (DWI)	-	H	-
F3 (P2)	MRI	4y 4mo		+	+	+ (T2, DWI)	+	H	-
F3 (P1)	MRI	10y 4mo		+	+	+ (T2, DWI)	+	H	-
F4 (P1)	MRI	2y 10mo		+	+	+ (DWI)	-	-	-
F5 (P1)	MRI	4y 2mo		+	-	-	-	-	B
	MRI	7y 6 mo		+	+	+ (T2, DWI)	+	H	S
F6 (P3)	MRI	17y 5mo		+	l	+ (T2, DWI)	+	I	-
F6 (P4)	CT	19y		+	I	-	-	-	I
F6 (P1)	MRI	11y 4mo		+	+	+ (T2)	+	I	-
	MRI	21 y 3mo		+	+	+ (T2)	+	H A	-
F6 (P2)	MRI	10y 4mo		+	+	+ (T2)	+	-	-
	MRI	13y 3mo		+	+	+ (T2)	+	-	-

+ Changes present; - changes absent

A = atrophy; B = changes borderline; DWI = diffusion-weighted MRI image; H = high (mild) T2- MRI signal in cerebral white matter; I = imaging quality or type not suitable to assess feature; S = Simple splenium(posterior) corpus callosum; T2 = T2-MRI sequence.

**Figure 3:**
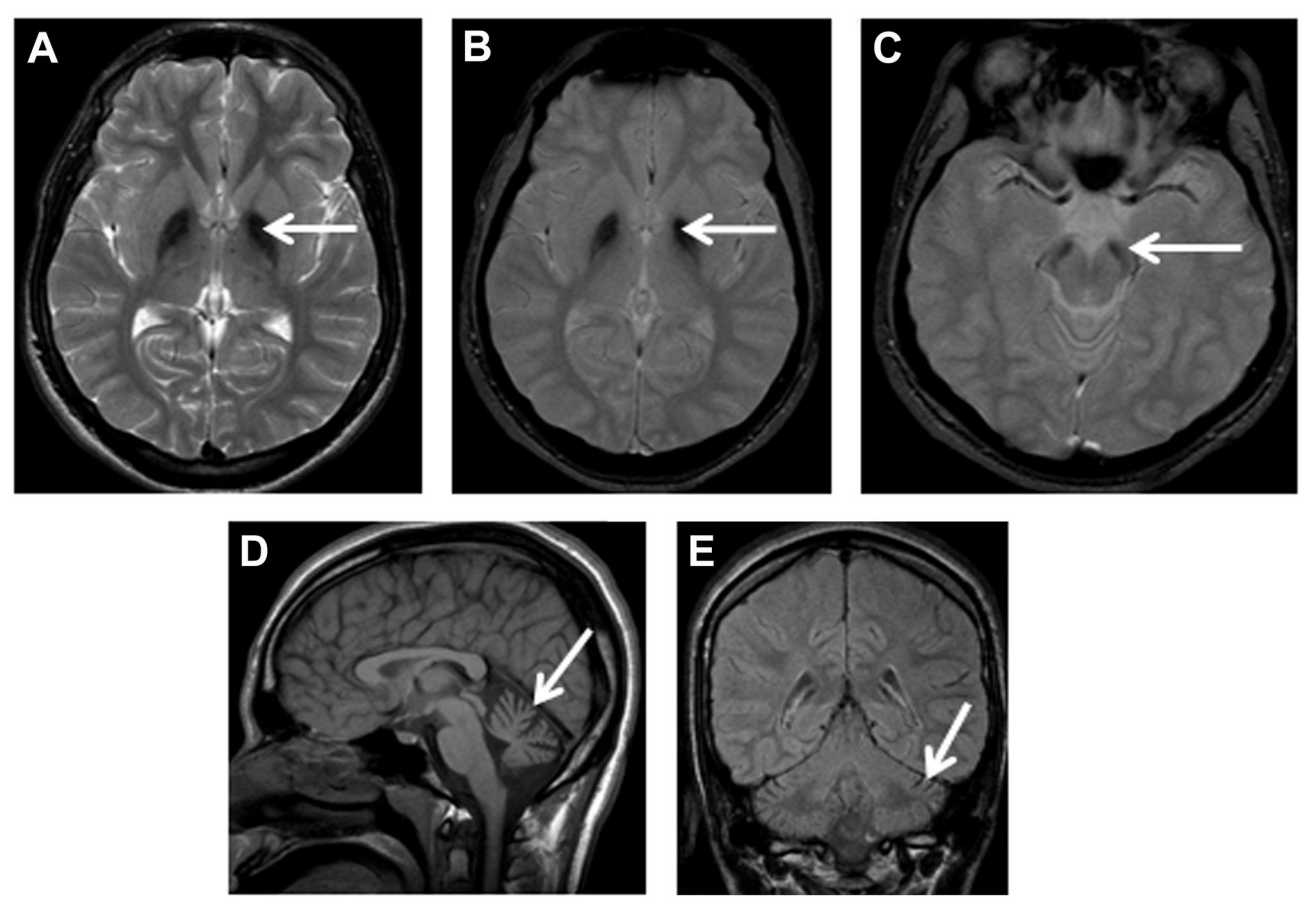
Patient F6 (P3) MRI, age 17 years 5 months. Axial T2-weighted (A), axial gradient (B and C), sagittal T1-weighted (D) and coronal fluid-attenuated inversion recovery (FLAIR, E) sequences highlight iron deposition as reduction in signal intensity in the globus pallidi (arrows in A and B) and *substantia* *nigra* (arrow in C). There is also cerebellar cortical atrophy with increased CSF spaces around the cerebellum (arrows in D and E).

**Figure 4:**
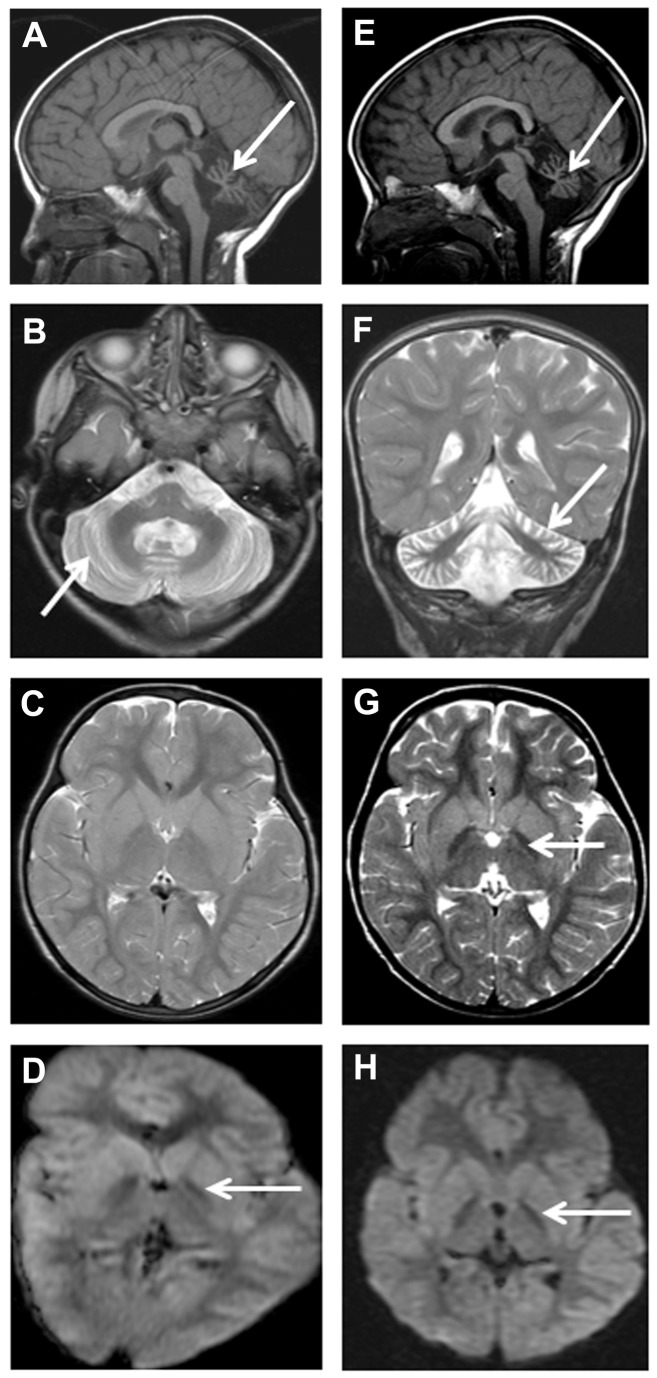
Patient F1 (P1) MRI, age 3 years 1 month (A-D), and 4 years 2 months (E-H). There is increased CSF space around the cerebellum (arrows in A and E) associated with cerebellar cortical atrophy and gliosis, with widening of folia and increased signal in the residual cerebellar cortex (arrows in B and F). Axial T2-weighted (C and G) and axial diffusion (D and H) highlight iron deposition as reduction in signal intensity in the *globus* *pallidi* in only the later T2-weighted sequence (G), but in both diffusion sequences (D and H).

Eight (80%) of the 10 children who had MRI scans showed evidence of increased iron deposition in the *globus pallidus*, seen as reduced signal on T2, FLAIR and/or diffusion weighted image (DWI) sequence in the initial scans (Figures 3 and 4). One child (F5 [P1]) (Figure 2) did not have the changes in the *globus pallidus* on the initial image performed at the age of 4.2 years, but showed it later at the age of 7.5 years. Another three children showed *globus pallidus* changes only on DWI at ages of 2.8 years (F4 [P1]), 3.1 years (F1 [P1]) and 3.3 years (F2 [P1]), respectively (Table 5). Repeated MRI, performed 13 months later in one of them (F1 [P1]) (Table 5 and Figure 4), showed the pallidal changes in both T2 and DWI sequences. An adolescent (F6 [P4]), who was subjected only to CT performed at 19 years of age, showed cerebellar atrophy with no evidence of *globus pallidus* changes (in the form of reduced density). His affected brother (F6 [P3]) showed clear features of brain iron deposition on MRI done at a younger age (17.4 years, Figure 3). Overall, the initial or only MRI scans, done for 10 patients at median age of 4.3 years (mean = 6.8 years; range = 1.3-17.4 years), revealed *globus pallidus* iron deposition in 8 (80%) whereas cerebellar atrophy was shown in all patients.

Increased iron in the *substantia nigra* was revealed by the initial MRI in 6 of 10 (60%) patients at a median age of 10.3 years (mean = 9.5 years; range = 3.1-17.4 years). Three of the patients with initial normal *substantia nigra* signal had their MRI at 1.3 year (F2 [P2]), 2.8 years (F4 [P1]), and 3.3 years (F2 [P1]) but no subsequent scans. The fourth patient (F5 [P1]), had his initial MRI at the age of 4.2 years, but showed *substantia nigra* iron deposition when imaging was repeated 3 years 4 months later (Table 5). With imaging allowing adequate assessment, 6 of 10 (60%) patients had cerebral white matter changes and one of them (F6 [P1]), also showed cerebral atrophy at the age of 21.3 years. Abnormality of corpus callosum was detected in 2 of 10 (20%) patients, at 4.2 years in the first one (F1 [P1]), and was borderline in the second (F5 [P1]) at the same age with abnormality detected 3 years and 4 months later. This consisted of simple appearance to the splenium which was thin and elongated. The initial MRI in the other patients showed normal corpus callosum (Table 5, Figures 3 and 4).

### Structural and functional analysis of muscle biopsies

**Figure 5:**
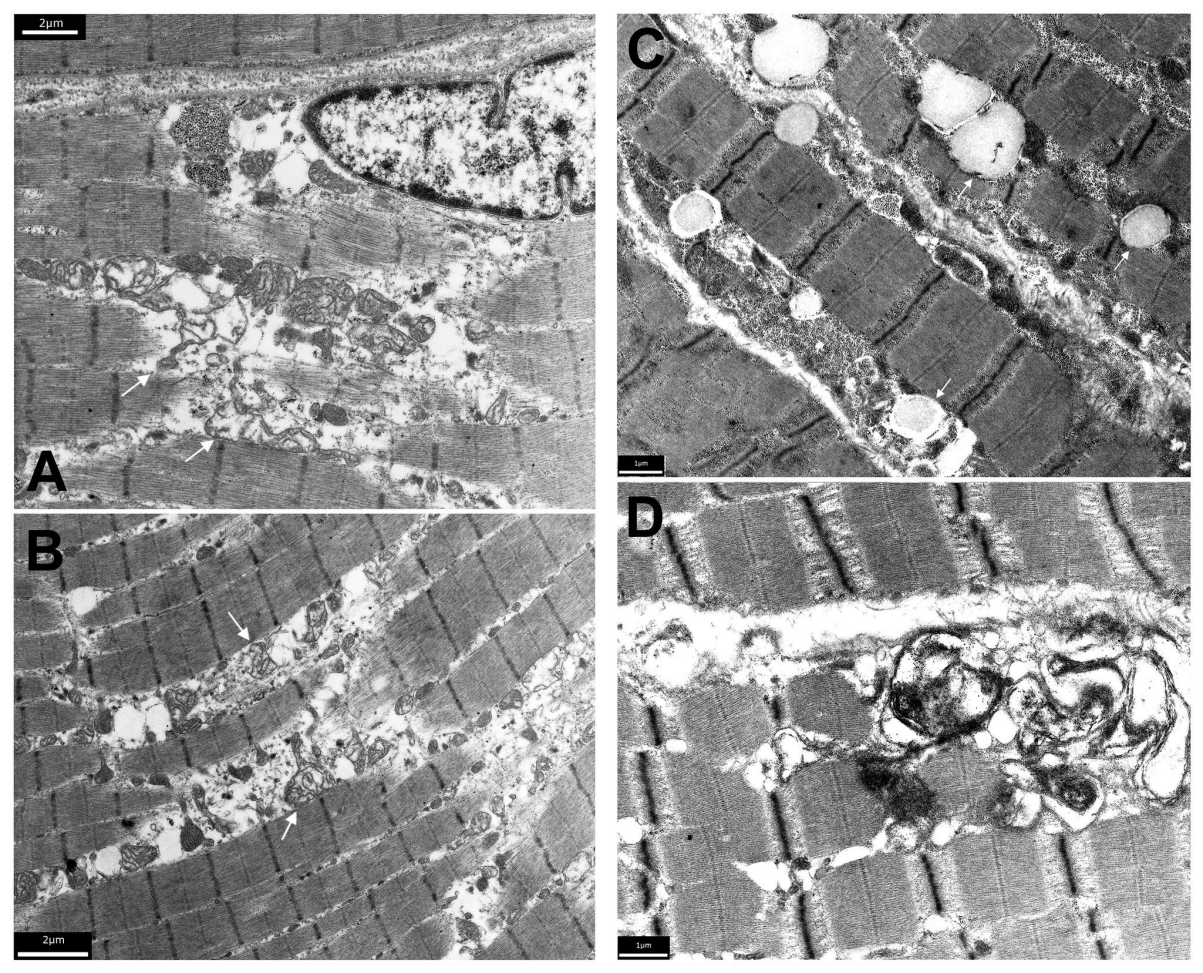
Electron microscopic alterations in the muscle biopsy of patient F6 (P1), age 12 years. (A and B) There is enlargement of the sarcoplasmic space between myofibrils associated with focal increase in granular and membranous material (arrows). The membranes are probably derived from the sarcoplasmic reticulum/myotubular system and from disintegrated mitochondria. There were no myonuclear inclusions. (C and D) Electron microscopic analysis of the muscle biopsy of patient F1 (P1), age 3 years. (C) Lipid droplets (arrows) in muscle fibres are moderately increased in size and number. (D) Subsarcolemmal accumulation of membranous material indicating proliferation of sarcoplasmic reticulum membranes.

Histological examination of muscle biopsies from 4 patients revealed non-specific neurogenic features. In contrast, electron microscopy (Figure 5) revealed significant abnormalities in the two examined muscle specimens. Alterations in the muscle biopsy (taken at the age of 12 years) of patient F6 (P1) with Karak syndrome phenotype (Table [1.1 and 2.1] and Figure 5) consisted of enlargement of the sarcoplasmic space between myofibrils associated with focal increase in granular and membranous material. There were no myonuclear inclusions. Biochemical assay of muscle enzymes of this patient [F6 (P1)] revealed a remarkably low activity of all mitochondrial enzymes measured. These included NADH: Q1 oxidoreductase (complex I) of 3.5 mU/mg (control range = 12.5-19.5), succinate: cyt *c* oxidoreductase (complex II + coenzyme Q + complex III) of 4.0 mU/mg (control range = 8.2-44), and decylubiquinol: cyt *c* oxidoreductase (complex III) of 41 mU/mg (control range = 54-434). Citrate synthase activity was at the lowest reference value (48 mU/mg; control range = 48-162). By contrast, the ratios between enzyme activity of the respiratory chain and citrate synthase were not remarkable. The relatively low activity of all enzymes on protein base, combined with the normal enzyme activity ratio’s, lead us to conclude that there is a relatively low mitochondrial content in the muscle of patient [F6 (P1)], rather than a deficiency of one or more respiratory chain enzymes. To confirm these results we determined the density of the intermyofibrillar mitochondria in the patient’s [F6 (P1)] biopsy and compared it to an age and gender matched healthy individual’s muscle specimen taken from the same location (*vastus lateralis* muscle) using electron microscopy. We found that the patient’s muscle showed a significant decrease in the mitochondrial density (0.255 +/- 0.05/µm^2^) in comparison to the control (0.379 +/- 0.06/µm^2^) (p=0.0001).

Electron microscopic analysis of the muscle biopsy (taken at the age of 3 years) of patient F1 (P1) [Table 1 and 2.1 and Figure 5] with INAD phenotype showed moderately increased lipid droplets in muscle fibres; both in size and numbers. There was also subsarcolemmal accumulation of membranous material indicating proliferation of sarcoplasmic reticulum membranes.

### Molecular genetics

**Figure 6:**
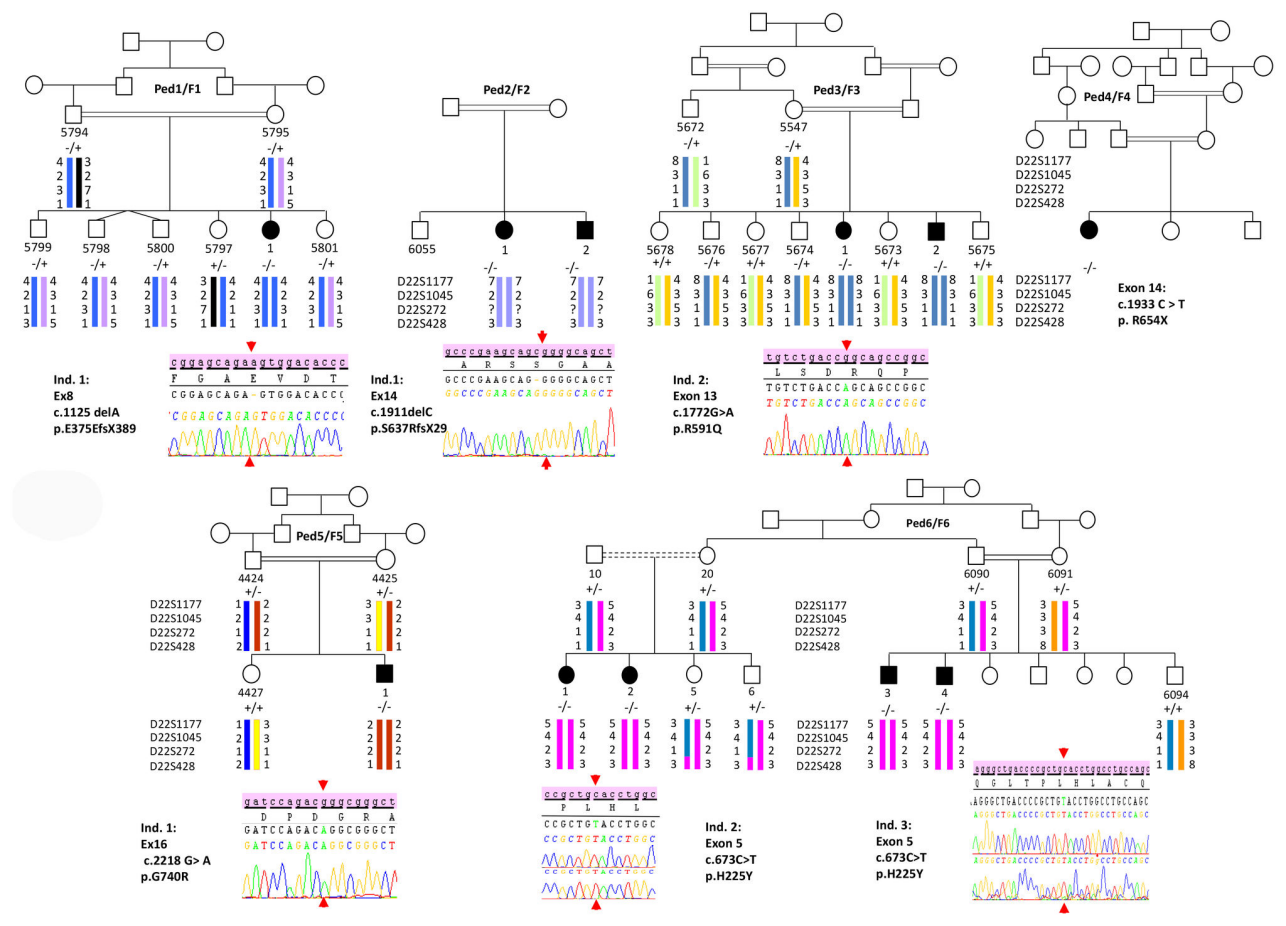
Genetics findings. Pedigrees (Ped) of the six Saudi Arabian families (F1-6) are represented in the same order as in the tables and in Figure 7. Patients were also numbered according to the Tables. Haplotypes were reconstructed manually (Family F4 was not subjected to genotyping) and chromatograms of each identified mutation are shown, except Family F4 which had direct sequencing in a private company. The segregation of the mutation, when possible, was shown for each pedigree with the corresponding symbols (“+”= wild type and “-“= mutated).

Genotyping allowed us to exclude the *PANK2* gene for 5 families while all the affected members of these were homozygous for the microsatellite markers flanking the *PLA2G6* locus (Figure 6). In accordance with the genotypes, pairwise and multipoint LOD scores were positive for each family and a maximum value of + 3.39 was reached in the largest pedigree (F6) [Table 6].

**Table 6:** Allegro software results of Multipoint LOD score values of four markers flanking the *PLA2G6* gene.

	Microsatellite Markers	D22S1177	D22S1045	D22S272	D22S428	
	relative position in cM	49.33	50.57	52.56	53.85	
Family	F1	0.0804	0.9257	**1.4307**	0.8841	
	F2	**1.3838**	1.2315	0.000	1.1293	
	F3	**2.1577**	1.0507	**2.1577**	1.9122	
	F5	**0.9309**	0.7270	0.7848	0.6854	
	F6	3.2045	1.5882	**3.3977**	-∞	
		LOD score values				

The maximum LOD score values for each family are indicated by bold characters,

Sequencing of the *PLA2G6* gene allowed us to identify six different homozygous mutations perfectly segregating with the disease in the corresponding family members (*n* = 35). All affected individuals were homozygous for the identified mutation while parents, for whom the DNA was available, were heterozygotes for the mutations and unaffected siblings were either heterozygotes or homozygous for the wild type allele (Figure 6). Five mutations, 2 missense (p.H225Y and p.G740R) and 3 truncating (p.E375EfsX389, p.S637RfsX29 and p.R654X) were newly identified. The additional missense mutation (p.R591Q) found in family F3 has already been reported [2,4]. The missense mutations p.H225Y and p.G740R were not found in at least 86 [range 86-175] North-African controls. Moreover, none of the six mutations we identified in this study was found in more than 5,352 controls from Exome variant Server (http://evs.gs.washington.edu/EVS/) (Table 7). The three truncating mutations (the nonsense one and the 2 insertions) were predicted to lead to the nonsense mediated mRNA decay (NMD) [Table 7]. The three missense mutations are all affecting well conserved amino-acids. However, p.H225Y mutation was predicted to be less severe (tolerated, possibly damaging) compared to p.R591Q and p.G710R that were predicted to be deleterious. In complementary DNA investigations to identify molecular events other than missense mutations causing-disease, MLPA detected no rearrangement in both *PLA2G6* and *PANK2* genes.

**Table 7:** Nature and position of the mutations identified in the patients homozygous for the *PLA2G6* locus markers.

Family	Exon	mutation		Tested in controls from Maghreb*	Exon Variant Server	Conservation across species	Computational Predictions	
		mRNA level	Protein level				SIFT ª	PolyPhen2^b^
F3	13	c.1772GA	p.R591Q	0/344	0/5357	26/27	Deleterious (0.03)	Probably damaging (1.000)
F5	16	c.2218GA	p.G740R	0/350	0/5235	20/27	Deleterious (0.00)	Probably damaging (1.000)
F6	5	c.673CT	p.H225Y	0/346	0/5354	25/27	Tolerated (0.17)	Possibly damaging (0.632)
F1	8	c.1125delA	p.E376WfsX14	0/172	0/5354			
F2	14	c.1911delC	p.S637RfsX29	Not done	0/5352			
F4	14	c.1933CT	p.R654X	Not done	0/5352			

The mutations were annotated according to the HGVS recommendations. In Bold, the reported mutation^4^. Prot. = protein.

Score values are in brackets, they are between 0 and 1 for SIFT and PolyPhen2 software’s.

* Results are reported in number of chromosomes. ^a^ SIFT score: the scores closer to 0 are the most deleterious. ^b^ Scores closer to 1 indicate that the mutation is damaging.

### Genotype Phenotype correlation

**Figure 7:**
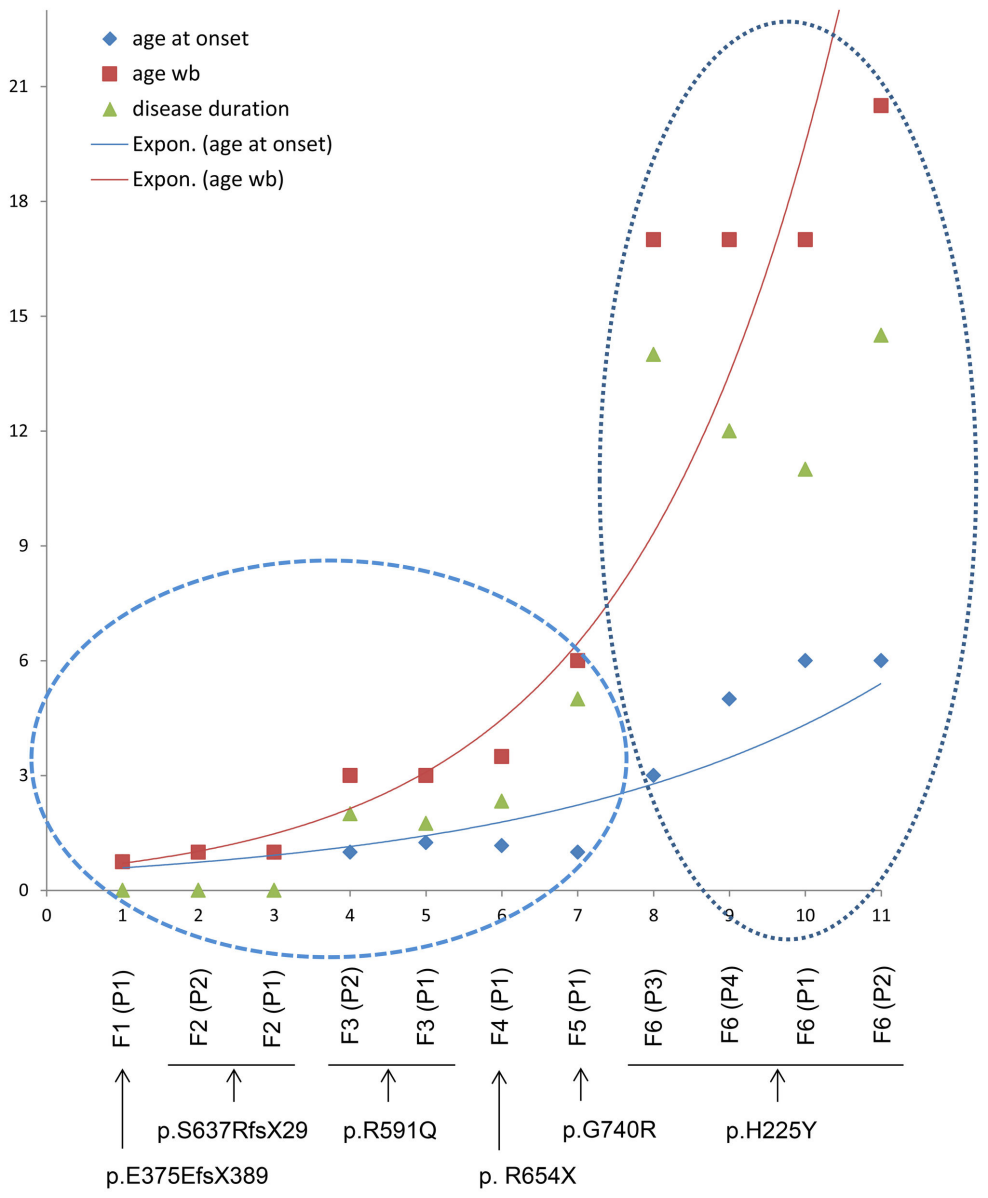
Genotype-phenotype correlation. Graphic representation of the evolution exponential tendency curves of the functional disability (red curve and red squares) and of the age at onset of ataxia (blue curve and blue squares) which both seem to depend on the nature of the mutation. Each number in X axis corresponds to one patient. Below this axis are indicated the codes of patients (from Families 1 to 6) and the corresponding mutation. Y axis corresponds to the age per years. Two groups are identified (dashed ellipses) depending on the age at onset of ataxia. The first one encompasses the patients with ataxia manifesting at or before 15 months of age, and the second one, patients with an onset between 3 and 6 years. The age when becoming wheelchair-bound (red squares) and the disease duration (green triangles) are most prominent clusters in the second group. Abbreviations: wb = wheelchair-bound. Expon = exponential.

In order to find whether the genotype influences the phenotype in our series of patients, we considered two relevant criteria for assessing the severity of the pathology at the functional disability level: (i) the age at onset of the disease manifesting as ataxia, and (ii) the evolution of the disease at the functional level. We expressed the results as a graph with exponential trend curves. A curve of the evolution of the age at onset of ataxia and a second one showing evolution of the age at which patients reach functional Stage 7 (wheelchair bound) were formulated. The disease duration was also considered in the graph (Figure 7). We could distinguish two groups of patients according to the age at onset and the age when patients reached the functional disability Stage 7. Group 1, whose age at onset was ≤ 15 months, reached Stage 7 very rapidly, and the disease duration before reaching Stage 7 was very short (zero to 5 years). Indeed, three patients (F1 [P1], F2 [P1] and [P2]) have never walked independently. The patient whose disease progression was slower (F5 [P1]), was wheelchair bound at 6 years, only 5 years after the onset of ataxia. This patient manifested atypical INAD [Table 1]. Group 1, which includes 7 patients (Figure 7), is located in the lower part of the curves. Group 2 consists of four patients from the same family (F6), all with Karak syndrome. The age at onset of ataxia in this group occurred between 3 and 6 years, at least 25 months later than the patient who manifested the disease latest in Group 1 (F3 [P1], at age 15 months). Two patients (F6 [P1 and P2]) in Group 2 started the disease at age 6 years, the same age when the patient with the slower disease progression in Group 1 (F5 [P1]) became wheelchair-bound. This group is in the exponential parts of the curves, reflecting on one hand a greater disease duration before reaching Stage 7 (11 to 14.5 years) and on the other hand the slower progression of the disease. In this group, there is also an interfamilial variability in terms of age at onset of ataxia, and pace of disease progression, even between the two sisters (F6 [P1 and P2]) whose disease started at age 6 years, but have reached a functional Stage 7 later in the second decade at 17 and 20.5 years, respectively. Interestingly, patients of this family are homozygous for the p.H225Y mutation predicted to be relatively less severe, unlike the other missense as well as the truncating mutations, in Group 1 (Table 7). The predicted damaging mutations are responsible for a severe phenotype characterized, at functional level, by a disease onset before the age of 16 months and a fast progression confining all patients to wheelchair before the age 6 years, either immediately after onset of ataxia (F1 [P1], F2 [P1 and P2]), or 5 years later (F5 [P1]). The other symptoms showed no correlation with the genotypes.

## Discussion

The present cohort represents one of the largest collections of patients from one country and the same ethnic background (Arabs) with neurodegeneration associated with *PLA2G6* mutations.

In the present study, 6 children (F1 [P1], F2 [P1 and P2], F3 [P1 and P2] and F4 [P1]) [Table 1] had classic INAD phenotype with onset within the first 2 years of life, hyperreflexia and tetraparesis (See Video S1) leading to loss of ambulation within 5 years [5,6], with ataxia being the earliest appearing symptom. A seventh patient (F5 [P1]) [Table 1], manifested atypical NAD with slower progression of the disease, ataxia and remarkable spasticity, and contracture deformities of the lower limbs which required surgical release. The phenotype of the remaining 4 patients from a single family (F6) [Table 1] is nearest to that of Karak syndrome [1]. Indeed, onset ranged between 3-6 years with ataxia, which was slowly progressive and associated with slow cognitive decline. Loss of ambulation was at the age of 17 years in 3 patients whereas the fourth walked until 20.5 years of age. The phenotype in the cohort we studied was relatively heterogeneous as compared to the homogeneous spectrum in 14 previously reported children [5]. Despite the varying phenotypes of the patients in the present study, ataxia was the constant and earliest appearing symptom at a median age of 14 months (mean = 29.6 months). This was supported by neuroimaging, which showed cerebellar cortical atrophy in all patients, even at the age of 15 months.

Two patients with INAD phenotype manifested psychiatric symptoms and three of 4 patients from family F6 (P1, P2 and P4) had phobias, panic attacks and/or bouts of rage. This could be explained by the involvement of the *locus caeruleus* in the disease, which has been documented pathologically in patients with *PLA2G6* mutations [4]. It is noteworthy that the *locus caeruleus*-noradrenergic system has been implicated in the pathogenesis of panic disorder, post-traumatic disorders, and several other psychiatric conditions [25-27].

In the present cohort, a female with Karak syndrome phenotype (F6 [P1]) developed complex partial seizure at 21 years of age, which was confirmed by EEG. Another 2 siblings with INAD had brief tonic seizures with normal EEG in one and non-specific changes in another. Fast rhythms on EEG were observed in 2 patients with classic INAD without clinical seizures [Table 3]. These fast rhythms are detected frequently on EEG in patients with INAD, and generalized seizures manifest in only a minority of cases [4-6,28,29].

Optic atrophy occurs in the majority of children with infantile and atypical NAD whereas nystagmus and strabismus are also common [4-6,28,29]. Saccadic eye movements were abnormal in the two described siblings with Karak syndrome, but fundoscopy revealed normal optic disks in both of them [1]. However, both patients are significantly younger than those described in the present report.

Neurophysiologic studies revealed features of distal axonal neuropathy in all 4 patients with Karak syndrome phenotype. Also 3 of 5 (60%) children with classic INAD had features of axonal neuropathy on NCS. This is similar to the findings in other cohort of patients with INAD [4,5].

Similar to previous observations, the cerebellar atrophy was the earliest and universal neuroimaging feature [4,5,28,29]. It can usually be detected by MRI after the age of 2 years [4,5,28,29] and even at a younger age by CT or MRI scans [5,28]. It’s noteworthy that genetic knockdown of *PLA2G6* in mice (iPLA2b-/-) was documented to lead to the development of cerebellar atrophy by the age of 13 months [30]. High signal intensity, indicating gliosis, in cerebellar cortex (as seen in 60% of the present cohort) is a frequent associated feature [4,5,28], although not universally detected with INAD phenotype [5,13,29,31]. Serial MRI in one patient in the present study confirmed previous observations showing that cerebellar gliosis subtly evolves with age and was associated with cerebral atrophy after the age of 20 years [5,28,29]. Simple splenium (posterior *corpus callosum* thinning) was evident in 2 patients (F1 [P1] and F5 [P1]) and borderline changes were detected initially in one of them (F5 [P1]). Similarly, one of 5 reported patients [28] who had INAD with identified mutations in *PLA2G6* gene, had thinning of the *corpus callosum* depicted by MRI at the age of 7 years. Also in a study on a cohort of children with *PLA2G6* gene mutations, all 12 patients with MRI scans of sufficient quality had abnormal corpus callosum with simple appearance to the splenium which was elongated, thin and slightly vertically oriented [5]. A total of 82% of patients had *globus pallidus* changes which were either initially present or became apparent on subsequent T2 and/or diffusion weighted imaging (DWI) MRI sequences. These changes were not apparent on CT done at 19 years of age for one patient (F6 [P4]) and at 4.2 years for another (F1 [P1]). The latter child had an earlier MRI at 3.1 years which revealed *globus pallidus* hypointensity on DWI only (Figure 4). From the present experience, DWI seems to precede T2-MRI sequence in revealing the pallidal changes, since DWI facilitated the detection of these alterations in another child (F4 [P1]) who had MRI at the age of 2.8 years (Table 5). T2 gradient MRI sequence is also more sensitive than T2 in depicting iron deposition in the *globus pallidus*, but was undertaken in only one patient (Figure 3). The eye-of-the-tiger sign, reported in the 2 patients with Karak syndrome [1] was not seen in any of the patients in this cohort. Similar to another study [5], longitudinal imaging data revealed iron deposition (manifesting as low signal/density) in the *substancia nigra* in all patients after the age of 5 years, and iron accumulation also seemed to become more appreciable later in the course of the disease [6].

The muscle biopsies were done in 4 patients to exclude mitochondrial disease and their standard histological characterization revealed non-specific neurogenic features. However, an unexpected feature of the study derives from muscle electron microscopy where focal increase in granular and membranous material was observed in 2 patients (F1 [P1] with classic INAD and F6 [P1] with Karak syndrome phenotype). This membranous material is probably derived from the sarcoplasmic reticulum/myotubular system and could have resulted from the disrupted membrane homeostasis following disruption of iPLA_2_-VIA encoded by *PLA2G6* gene. iPLA2 is essential for maintenance and repair of cellular membranes because of its requirement for phospholipid remodelling [32]. To the best of our knowledge, the ultrastructural findings described here have not been reported previously in cases of neurodegeneration associated with *PLA2G6* mutations. On the other hand, biochemical analysis of the muscle biopsy of a patient with Karak syndrome phenotype (F6 [P1], taken at the age of 12 years) showed a relatively low mitochondrial content in the muscle. This is compatible with the observed ultrastructural abnormalities seen by electron microscopy, and is also in agreement with the proposed role of PLA2G6 in protecting the mitochondrial membrane from peroxidation [33].

This is the first time that such a genotype phenotype correlation was highlighted in a cohort of PLAN patients, even in studies which included a group of patients belonging to the same ethnic background. Indeed, in the reported series [5], there was no evidence of genotype phenotype correlation for the same mutation (p.K545T) which has been identified at homozygous state in 9 out of 11 Pakistani patients. In the present study, apart from the onset of ataxia and evolution of functional disability, phenotypic variability has also manifested in all other evaluated clinical symptoms and signs. This demonstrates that other factors are involved in modulation of the variability of the broad clinical spectrum of PLAN.

In conclusion, we describe the phenotypic and genetic spectrum of 11 patients followed for a maximum period of 17 years and report six underlying *PLA2G6* gene mutations, five of which are novel. We have shown that the phenotype of neurodegeneration associated with *PLA2G6* mutations is variable in this cohort of patients who belong to the same ethnic background but, in terms of functional disability, was influenced by the genotype. Nevertheless, cerebellar atrophy is the constant and earliest feature of the disease, and precedes brain iron accumulation, leading to the provisional diagnosis of a recessive progressive ataxia in these patients. It is noteworthy that variable combinations of sites of degeneration and associated symptoms are often observed in childhood-onset recessive ataxias including epilepsy and abnormal cognition [34-36]. Electron microscopy showed, in 2 patients, an unexpected feature of focal increase in granular and membranous material probably derived from the sarcoplasmic reticulum/myotubular system. Biochemical analysis of the muscle biopsy of a patient showed a relatively low mitochondrial content in the muscle. This other new finding is compatible with the ultrastructural abnormalities seen by EM, and is also in agreement with the proposed role of PLA2G6 in protecting the mitochondrial membrane from peroxidation. Molecular testing for *PLA2G6* mutations is, therefore, indicated in childhood-onset ataxia syndromes, if neuroimaging shows cerebellar atrophy with or without evidence of brain iron accumulation. Considering the age at onset and the functional disability, there is an evidence of genotype-phenotype correlation. However, the wide intra- and inter-familial variability of the disease in physiological, psychiatric and other clinical aspects, cannot be explained only by single homozygous mutations in the *PLA2G6* gene. At least, other environmental and/or genetic factors (other variants in the genome, epigenetics, etc.) might probably modulate the disease presentation.

## Supporting Information

Video S1 **Patient F2 (P2) with INAD, aged 2.4 years, showing tetraparesis and brisk reflexes (including adductor reflex) despite absence of leg stiffness (note the frog position of the lower limbs).**
(WMA)

## References

[1] Mubaidin A, Roberts E, Hampshire D, Dehyyat M, Shurbaji A, et al. (2003) Karak syndrome: novel degenerative disorder of the basal ganglia and cerebellum. J Med Genet 40: 543-546. doi:10.1136/jmg.40.7.543. PubMed: 12843330. 12843330 PMC1735513

[2] Morgan NV, Westaway SK, Morton JE, Gregory A, Gissen P, et al. (2006) PLA2G6, encoding a phospholipase A2, is mutated in neurodegenerative disorders with high brain iron . Nat Genet. 38(7): 752-54754. doi:10.1038/ng1826. PubMed: 16783378. 16783378 PMC2117328

[3] Khateeb S, Flusser H, Ofir R, Shelef I, Narkis G, et al. (2006) PLA2G6 mutation underlies infantile neuroaxonal dystrophy. Am J Hum Genet 79: 942-948. doi:10.1086/508572. PubMed: 17033970. 17033970 PMC1698558

[4] Gregory A, Westaway SK, Holm IE, Kotzbauer PT, Hogarth P, et al. (2008) Neurodegeneration associated with genetic defects in phospholipase A2. Neurology 71:1042-1049. 10.1212/01.wnl.0000327094.67726.28 PMC2676964 18799783

[5] Kurian MA, Morgan NV, MacPherson L, Foster K, Peake D, et al. (2008) Phenotypic spectrum of neurodegeneration associated with mutations in the PLA2G6 gene (PLAN). Neurology 70: 1623-1629. doi:10.1212/01.wnl.0000310986.48286.8e. PubMed: 18443314. 18443314

[6] Gregory A, Polster BJ, Hayflick SJ (2009). Clinical and genetic delineation of neurodegeneration with brain iron accumulation. J Med Genet 46:: 73-80. PubMed: 18981035. 18981035 10.1136/jmg.2008.061929 PMC2675558

[7] XuWu Y, Jiang Y, Gao Z, Wang J, Yuan Y, et al. (2009) Clinical study and PLA2G6 mutation screening analysis in Chinese patients with infantile neuroaxonal dystrophy. Eur J Neurol 16: 240-245. doi:10.1111/j.1468-1331.2008.02397.x. PubMed: 19138334. 19138334

[8] Sina F, Shojaee S, Elahi E, Paisán-Ruiz C (2009) R632W mutation in PLA2G6 segregates with dystonia-Parkinsonism in a consanguineous Iranian family. Eur J Neurol 16: 101-104. doi:10.1111/j.1468-1331.2008.02356.x. PubMed: 19087156. 19087156

[9] Paisán-Ruiz C, Guevara R, Federoff M, Hanagasi H, Sina F, et al. (2010) Early-onset L-dopa-responsive parkinsonism with pyramidal signs due to ATP13A2, PLA2G6, FBXO7 and spatacsin mutations. Mov Disord 25(12): 1791-8001800. doi:10.1002/mds.23221. PubMed: 20669327. 20669327 PMC6005705

[10] Bower MA, Bushara K, Dempsey MA, Das S, Tuite PJ (2011) Novel mutations in siblings with later-onset PLA2G6**-**associated neurodegeneration (PLAN). Mov Disord 26: 1768–691769. doi:10.1002/mds.23626. PubMed: 21520282. 21520282

[11] Yoshino H, Tomiyama H, Tachibana N, Ogaki K, Li Y, et al. (2010) Phenotypic spectrum of patients with *PLA2G6* mutation and PARK14-linked parkinsonism. Neurology 75: 1356–611361. doi:10.1212/WNL.0b013e3181f73649. PubMed: 20938027. 20938027

[12] Lu CS, Lai SC, Wu RM, Weng YH, Huang CL, et al. (2012) PLA2G6 mutations in PARK14-linked young-onset parkinsonism and sporadic Parkinson’s disease. Am J Med Genet Part B 159B: 183–191. doi:10.1002/ajmg.b.32012. PubMed: 22213678. 22213678

[13] Tonelli A, Romaniello R, Grasso R, Cavallini A, Righini A, et al. (2010) Novel splice-site mutations and a large intragenic deletion in PLA2G6 associated with a severe and rapidly progressive form of infantile neuroaxonal dystrophy. Clin Genet 78(5): 332-409. PubMed: 20584031. 10.1111/j.1399-0004.2010.01417.x 20584031

[14] Balsinde J, Balboa MA (2005) Cellular regulation and proposed biological functions of group VIA calcium independent phospholipase A2 in activated cells. Cell Signal 17: 1052-1062. doi:10.1016/j.cellsig.2005.03.002. PubMed: 15993747. 15993747

[15] Shinzawa K, Sumi H, Ikawa M, Matsuoka Y, Okabe M, et al. (2008) Neuroaxonal dystrophy caused by group VIA phospholipase A2 deficiency in mice: a model of human neurodegenerative disease. J Neurosci 28: 2212-2220. doi:10.1523/JNEUROSCI.4354-07.2008. PubMed: 18305254. 18305254 PMC6671850

[16] Oh J (1993). Clinical electromyography: nerve conduction studies. 2nd edition. Williams & WilkinsBaltimore, : Baltimore Williams & Wilkins.

[17] GarciaGarcía A, Calleja J, Antolín FM, Berciano J (2000) Peripheral motor and sensory nerve conduction studies in normal infants and children . Clin Neurophysiol 111: 513-520. doi:10.1016/S1388-2457(99)00279-5. PubMed: 10699415. 10699415

[18] Kimura J (2001). Electrodiagnosis in diseases of nerve and muscle: Principles and practice. New York: Oxford University Press pp. p. 109,

[19] Preston DC, Shapiro BE (2005) Electromyography and neuromuscular disorders: Clinical-electrophysiologic correlations. 2nd edition. Portland: . Butterworth-Heinemann pp. 628-629.

[20] Weis J, Dimpfel W, Schröder JM (1995) Nerve conduction changes and fine structural alterations of extra- and intrafusal muscle and nerve fibers in streptozotocin diabetic rats. Muscle Nerve. 22: 175-84184. PubMed: 7823975. 10.1002/mus.880180205 7823975

[21] Grosser HR, Hesselink MK, Duimel H, Ward KA, Schols AM (2007) Reduced mitochondrial density in the varus laterals muscle of patients with COPD. Eur. Respir J. 1: 73-979. 10.1183/09031936.00146906 17428811

[22] Fischer JC, Ruitenbeek W, Gabreëls FJ, Janssen AJ, Renier WO, et al. (1986) A mitochondrial encephalomyopathy: the first case with an established defect at the level of coenzyme Q. Eur J Pediatr 144: 441-444. doi:10.1007/BF00441735. PubMed: 3956532. 3956532

[23] Sperl W, Ruitenbeek W, Kerkhof CM, Sengers RC, Trijbels JM, et al. (1990) Deficiency of the a and b subunits of pyruvate dehydrogenase in a patient with lactic acidosis and unexpected sudden death. Eur J Pediatr 149: 487-492. doi:10.1007/BF01959401. PubMed: 2189731. 2189731

[24] Gudbjartsson DF, Jonasson K, Frigge ML, Kong A (2000). Allegro, a new computer program for multipoint linkage analysis. Nat Genet 25: 12-13. doi:10.1038/75514. PubMed: 10802644. 10802644

[25] Samuels ER, Szabadi E (2008) Functional neuroanatomy of the noradrenergic locus coeruleus: It’s roles in the regulation of arousal and autonomic functionPart II: Physiological and pharmacological manipulations and pathological alterations of locus coeruleus activity in humans. Curr Neuropharmacol 6: 254-285. doi:10.2174/157015908785777193. PubMed: 19506724. 19506724 PMC2687931

[26] Mehler MF, Purpura DP (2009) Autism, fever, epigentics and the locus coeruleus. Brain . Res Rev 59: 388-92392. doi:10.1016/j.brainresrev.2008.11.001. PMC2668953 19059284

[27] Itoi K, Sugimoto N (2010) the brainstem noradrenergic systems in stress, anxiety and depression. J Neuroendocrinol 22: 355-361. doi:10.1111/j.1365-2826.2010.01988.x. PubMed: 20210846. 20210846

[28] Carrilho I, Santos M, GuimaraesGuimarães A, Teixeira J, Chorão R, et al. (2008) Infantile neuroaxonal dystrophy: what’s most important for the diagnosis? Eur J Paediatr Neurol 12: 491–500. doi:10.1016/j.ejpn.2008.01.005. PubMed: 18359254. 18359254

[29] Wu Y, Jiang Y, Gao Z, Wang J, Yuan Y, et al. (2009) Clinical study and PLA2G6 mutation screening analysis in Chinese patients with infantile neuroaxonal dystrophy. Eur J Neurol 16: 240–245. doi:10.1111/j.1468-1331.2008.02397.x. PubMed: 19138334. 19138334

[30] Zhao Z, Wang J, Zhao C, Bi W, Yue Z, et al. (2011) Genetic Ablation of PLA2G6 in Mice Leads to Cerebellar Atrophy Characterized by Purkinje Cell Loss and Glial Cell Activation. PLoOS ONE 6(10): e26991. doi:10.1371/journal.pone.0026991. PubMed: 22046428. PMC3203935 22046428

[31] Biancheri R, Rossi A, Alpigiani G, Filocamo M, Gandolfo C, et al. (2007) Cerebellar atrophy without cerebellar cortex hyperintensity in infantile neuroaxonal dystrophy (INAD) due to PLA2G6 mutation. Eur J Paediatr Neurol 11: 175–177. doi:10.1016/j.ejpn.2006.11.013. PubMed: 17254819. 17254819

[32] Lands WE. (1960) Metabolism of glycerolipids-2. The enzymatic acylation of lysolecithin. J Biol Chem, 235 : 2233-2237. PubMed: 14413818. 14413818

[33] Zhao Z, Zhang X, Zhao C, Choi J, Shi J et al. (2010) Protection of pancreatic beta-cells by group VIA phospholipase A(2)-mediated repair of mitochondrial membrane peroxidation. Endocrinology. 151(7): 3038-483048. Erratum in Endocrinology 2011 152(1): 336 doi:10.1210/en.2010-0016. PubMed: 20463052. 20463052 PMC2903934

[34] Salih MA, Ahlsten G, StalbergStålberg E, Schmidt R, Sunnegårdh J, et al. (1990) Fredreich’s ataxia in 13 children: presentation and evolution with neurophysiologic, electrocardiographic, and echocardiographic features. J Child Neurol 5:: 321-326. doi:10.1177/088307389000500410. PubMed: 2174072. 2174072

[35] Assoum M, Salih MAM, Drouot N, H’Mida-Ben Brahim D, Lagier-Tourenne C, et al. (2010) Rundataxin, a novel protein with RUN and diacylglycerol binding domains, is mutant in a new recessive ataxia. Brain 133(8): 2439-2447. doi:10.1093/brain/awq181. 20826435

[36] Assoum M, Salih MA, Drouot N, Hnia K, Martelli A, et al (2013) The Salih ataxia mutation impairs Rubicon endosomal localization. The Cerebellum, Jun 1 [Epub ahead of print]. PubMed: 23728897. 10.1007/s12311-013-0489-4 23728897

