# Correction: New Findings in a Global Approach to Dissect the Whole Phenotype of *PLA2G6* Gene Mutations

**DOI:** 10.1371/annotation/cb01a74a-3330-4412-8040-2a94842420ed

**Published:** 2013-11-06

**Authors:** Mustafa A. Salih, Emeline Mundwiller, Arif O. Khan, Abdulmajeed AlDrees, Salah A. Elmalik, Hamdy H. Hassan, Mohammed Al-Owain, Hisham M. S. Alkhalidi, Istvan Katona, Mohammad M. Kabiraj, Roman Chrast, Amal Y. Kentab, Hamad Alzaidan, Richard J. Rodenburg, Thomas M. Bosley, Joachim Weis, Michel Koenig, Giovanni Stevanin, Hamid Azzedine

There were errors in the Funding section. The correct funding information is as follows:

MAS and co-authors are thankful to the Deanship of Scientific Research, King Saud University, Riyadh, Saudi Arabia, for supporting the work through the research group project number RGP-VPP-301. RC was supported by the Gebert Rüf Foundation (grant number GRS-046/09). GS was supported by the French National Agency for Research(ANR), Association Contre les Syndromes Cérébelleux, France, The Verum Foundation, the European Union (Omics call, "Neuromics"), The Fondation Roger de Spoelberch and the program "Investissements d'avenir" ANR-10-IAIHU-06 (to the Brain and Spine Institute). HA was supported by Association Française contre les Myopathies (AFM), France. The funders had no role in study design, data collection and analysis, decision to publish, or preparation of the manuscript. 

